# Effectiveness of Surgical Approach of Insertion Ventilation Tubes (Tympanostomy) and Adenoidectomy in Comparison with Non-Surgical Approach (Watchful Waiting Approach) in Children at the Age between 1 and 6 and Who Suffer from Otitis Media with Effusion (OME) in 12-Month Period of Observation—The Retrospective Analysis

**DOI:** 10.3390/ijerph182312502

**Published:** 2021-11-27

**Authors:** Magdalena Beata Skarzynska, Elżbieta Gos, Natalia Czajka, Milaine Dominici Sanfis, Piotr Henryk Skarzynski

**Affiliations:** 1 Institute of Sensory Organs, Nadarzyn, 05-830 Warsaw, Poland; p.skarzynski@csim.pl; 2 Center of Hearing and Speech Medincus, Kajetany, 05-830 Warsaw, Poland; 3 Department of Teleaudiology and Screening, World Hearing Center, Institute of Physiology and Pathology of Hearing, Kajetany, 05-830 Warsaw, Poland; e.gos@ifps.org.pl (E.G.); n.wasilewska@ifps.org.pl (N.C.); 4 Child and Adolescent Heath Program, Faculty of Medical Sciences, University of Campinas, Campinas 13083-970, Brazil; msanfins@uol.com.br; 5 Heart Failure and Cardiac Rehabilitation Department, Faculty of Medicine, Medical University of Warsaw, 03-242 Warsaw, Poland

**Keywords:** otitis media with effusion, adenoidectomy, ventilation tubes

## Abstract

(1) Background: Otitis media with effusion (OME) is one of the most common diseases in childhood. The objective was to assess clinically the effectiveness of the surgical approach (tube insertion with adenoidectomy) in comparison with the non-surgical approach (watchful waiting) during a 12-month observation period. (2) Methods: This study was retrospective and obtained approval from the bioethics committee. The criteria of inclusion in the first group (surgical approach) were: (1) a diagnosis of chronic otitis media with effusion in children aged between 1 and 6 years; (2) their medical history showed that they had undergone adenoidectomy and tympanostomy with the insertion of ventilation tubes (VTs). The criteria for inclusion in the second group (non-surgery) were similar to the first group except that their medical history showed they had not undergone adenoidectomy or tympanostomy with the insertion of VTs. There were 422 children included in the surgical group and 50 children in the non-surgical group, and the period of observation was 12 months. (3) Results: For the entire surgical group, the number of healthy days ranged from 20 to 365, with a mean of 328.0 days (SD = 91.4).In the non-surgical group, the number of healthy days ranged from 13 to 365, with a mean of 169.2 days (SD = 127.3). The difference in the number of healthy days was statistically significant (*p* < 0.001). The certainty of treatment in the first group was higher than in the second group, and the number of days without recurrence was significantly higher than in the second group. In the first group, there were 71 recurrences from 422 children (16.8%), and, in the second subgroup, there were 40 recurrences of acute otitis media (AOM) from 50 children (80%). The RR was 0.21. (4) Conclusions: The surgical approach in children aged 1–6 years who have been diagnosed with otitis media with effusion is reasonable and beneficial for the child.

## 1. Introduction

Otitis media with effusion (OME) is one of the most common diseases in childhood. The condition refers to an accumulation of fluid in the cavity of the middle ear behind an intact membrane and without any symptoms of acute infection such as fever, ear pain, headache, irritability, or pulling on the ears [1]. The Eustachian tube may also be more or less blocked. Some 70% of children have had OME at least once by 2 years old and 80% by age 4 [2,3]. OME involves the accumulation of thick mucoid fluid or thin serous fluid in the middle ear cavity, and the fluid adversely affects the efficiency with which sound is transferred from the eardrum to the cochlea via the three auditory ossicles. This loss of efficiency leads to conductive hearing loss, which in turn can delay language and speech development and finally lead to impaired learning [4,5]. A clinically effective treatment for OME is a “watchful waiting” strategy over the initial 3 months, allowing the condition to spontaneously resolve. Only in cases where the problem persists may surgery be required [6]. A range of research findings show how important it is to identify children with hearing disorders and implement appropriate treatment and rehabilitation early on [7,8,9]. Childhood hearing screening in school children should be the primary tool for identifying hearing loss that was not recognized at birth or that developed later [10,11,12]. The symptoms of hearing loss in children can manifest in a variety of ways and, in some cases, may go unnoticed [13]. A study conducted by Lo et al. [14] among parents of children with OME shows very low sensitivity: only 12% for predicting positive audiometric results. The high prevalence of otitis media, especially with effusion, was identified as the most common cause of hearing loss in children in a multinational study of 5776 children in Germany, Italy, Spain, Sweden, and the UK [15]. The prevalence of acute otitis media was 256/1000 person-years during the prospective study period. The incidence was lowest in Italy (195, 95% CI 171–222) and highest in Spain (328, 95% CI 296–363). The study conducted by the Institute Physiology and Pathology of Hearing in Poland indicated that otitis media with effusion was found in 37% of children with abnormal otoscopy [16,17]. In a study conducted in Lagos, Nigeria, low-frequency hearing loss (LFHL) was found in 48.6% of impaired ears [18]. One of the most common causes of LFHL was otitis media with effusion, tympanosclerosis, and fungal otitis. In terms of comparison, data from a U.S. population study reported a much lower rate of LFHL (7.1%). Recent similar data were obtained in a screening study in Tajikistan, where 34% of the children screened were found to have LFHL [19]. 

OME, sometimes referred to as otitis media secretoria, is traditionally classified into three categories: acute otitis media with effusion (AOM, with symptoms lasting up to 10 days), subacute otitis media with effusion (between 10 days and 3 months), and chronic otitis media with effusion (above 3 months) [20]. The commonest reasons for a diagnosis of OME are: acute otitis media (AOM), the presence of a negative pressure in the tympanic cavity, allergy, *nasopharyngititis*, infection caused by *Alloiococcus otitidis,* biofilm, craniofacial dystrophy, gastroesophageal reflux, tonsil hypertrophy, and poor pneumatization of the mastoid process [21]. The symptoms of OME include conductive hearing loss (usually bilateral), feeling of water in the ear, lack of fever, and otalgia. Hearing loss is between 20 and 60 dB. According to current guidelines, pharmacological treatment with decongestants, intranasal corticosteroids, and antihistamines is either ineffective or may, in some cases, cause adverse reactions [22,23,24]. The guidelines therefore recommend a 3-month period (watchful waiting) in children with OME who are at no risk of impaired language, speech, or learning development [25].

Tympanostomy with insertion of ventilation tubes (VTs) is the commonest surgical treatment for OME [26]. VTs are recommended for children with persistent OME and with documented hearing difficulties following a 3-month watchful waiting period [25,27]. Insertion of VTs effectively overcomes the problem in children who have recurrent AOM. As an adjunct to the insertion of VTs, adenoidectomy is beneficial in affected children aged 4 or older [28,29]. VTs are classified as a medical device and are placed in the tympanic membrane under general anesthesia. The tube usually remains in the eardrum between 6 and 12 months, before eventually falling out by itself. The size and type needs to be adjusted depending on how the disease is advancing and the condition of the tympanic membrane. In addition, the size and anatomical condition of any middle ear implant are also important factors. For children with bilateral OME where VT insertion is unacceptable or not recommended, current guidelines indicate that an adjuvant adenoidectomy is a possible option [29].

### Objective

The objective of this analysis is to clinically assess the effectiveness of the surgical approach (tube insertion with adenoidectomy) in comparison with the non-surgical approach (watchful waiting) during a 12-month observation period. 

## 2. Materials and Methods

This study was retrospective and obtained approval from the bioethics committee (IFPS:KB/Oświadczenie nr 16/2021r). The criteria of inclusion in the first group (surgical approach) were: (1) a diagnosis of chronic otitis media with effusion; (2) age of diagnosis between 1 and 6 years; (3) their medical history showed that they had undergone adenoidectomy and tympanostomy with the insertion of VTs. The criteria for inclusion in the second group (non-surgery) were similar to the first group except that their medical history showed they had not undergone adenoidectomy or tympanostomy with the insertion of VTs. There were 422 children included in the surgical group and 50 children in the non-surgical group. The starting point for data collection was different in the two groups. In the first group, the beginning of data collection was the date of surgery and the medical history was analyzed from this point. In the second group, the beginning of data collection was the day that they were diagnosed with chronic otitis media with effusion. In both groups, data were collected for 12 months. The difference in the number of children in the groups was due to a difficulty in collecting enough data from the second group. The institute where the data were collected was a quaternary care hospital, where otorhinolaryngologic surgeries were common, and so the number of children having a 12-month history of no surgery was low. 

## 3. Results

There were 422 children who were diagnosed with acute otitis media. Those who underwent surgical treatment constituted the study group, and children who were treated conservatively were the control group. The study group consisted of 422 children, 1 to 6 years old (mean 4.05 years, SD = 0.96); there were 25 boys (59.2%) and 172 girls (40.8%). The control group comprised 50 children, made up of 26 boys (52%) and 24 girls (48%); their ages ranged from 1.8 to 6.0 years old, and the mean age was 3.65 (SD = 1.02). In both groups, most children were 3–4 years old. Table 1 provides data about their ages and Table 2 provides data about the sex distribution; Table 3 gives the age distributions. 

In the first group (surgical), there were 189 children (44.8%) who had upper respiratory tract infections and 55 (13.0%) who had recurrent acute otitis media in their medical history. In the non-surgical group, there was a similar percentage (44%) who had upper respiratory tract infections and 68% (34 patients) with recurrent acute otitis media. In both groups together, 18.9% had a history of recurrent acute otitis media and 44.7% had upper respiratory tract infections (Table 4 and Table 5).

The number of ENT specialist consultations related to otitis media prior to surgery ranged from 1 to 9; the mean of the study group was 1.46 (SD = 1.02). Most children (*n* = 312; 73.9%) had one consultation, 63 (14.9%) had two consultations, 29 (6.9%) had three consultations, and 18 (4.2%) had more than three (Table 6). 

Both groups of children were observed for a 12-month follow-up period. In the surgery group, the observation period started from the day of operation and finished 12 months later. In the non-surgical group, observations started from the day of diagnosis of OME and finished 12 months later. In both groups, the number of cases of AOM during this period was monitored and calculated (Table 7). It can be seen that in the surgical group, the number of AOM cases was only 0.22 on average, while, in the non-surgical group, it was 1.16.

The surgical treatment was deemed to have failed if a child experienced a recurrence of AOM in the 12 months after surgery. The outcome measure was intervention failure, and was recorded as the number of healthy days before AOM recurred. At the 12-month follow-up, it was found that 351 children (83.2%) did not have any recurrence of AOM during the 12 months after surgery, while 72 children (17%) experienced a recurrence of AOM in that time. Of children having AOM recurrence, 51 had one AOM episode, 17 children had two episodes, and three children had three AOM episodes (Table 7).

**Figure 1:**
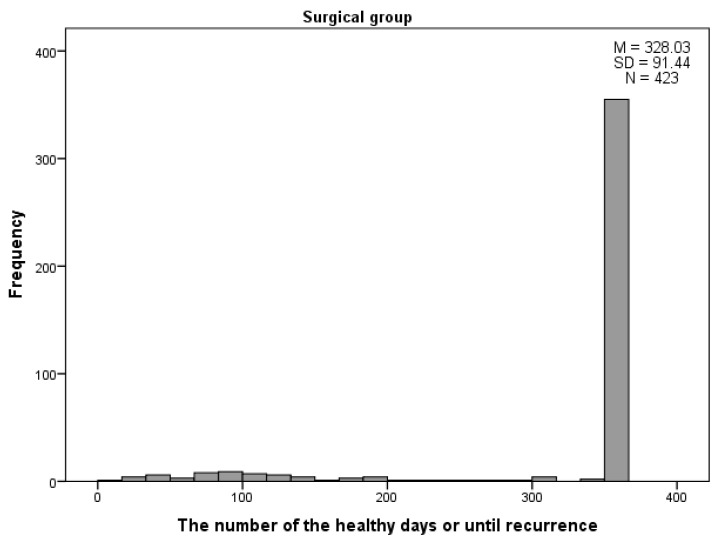
The number of healthy days within 12-month follow-up period after surgery in the surgical group.

**Figure 2:**
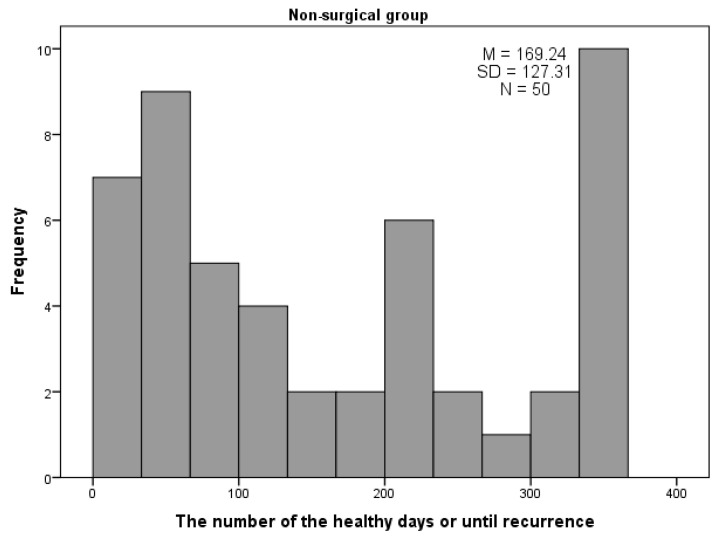
The number of healthy days within 12-month follow-up period in the non-surgical group.

For the surgical group (*n* = 422), the number of healthy days ranged from 20 to 365, with a mean of 328.0 days (*SD* = 91.4) (Figure 1). In the second group (non-surgical), the number of healthy days ranged from 13 to 365, with a mean of 169.2 days (SD = 127.3) (Figure 2). The difference between the number of healthy days was statistically significant (*p* < 0.001).

In the non-surgical group, the effect of treatment was more diverse than in the surgical group. In the non-surgical group, there were 10 children who lasted for one year (365 days) without any recurrence, but there was also roughly the same number (*n* = 7) who had reached less than 30 days without recurrence, and nine who were healthy for no more than 60 days. The certainty of treatment in the first group (surgical) was higher than in the second group, and also the number of days without recurrence was significantly higher than in the second group (Table 8).

The risk ratio (RR) was measured by comparing the recurrence of needing an ENT consultation in one group (the surgical group) with the non-surgical group. In the first group, there were 71 recurrences from 422 children (16.8%), and in the second subgroup, there were 40 recurrences of AOM from 50 children (80%). The RR was calculated according to the formula RR = (71/422)/(40/50) = 0.1682/0.80, with the result of 0.21. RR was also calculated in MedCalc (*p* < 0.0001) (Table 9). 

### Evaluation of Pharmacological Treatment in the Non-Surgical Group

Children in the non-surgical group were treated with antibiotics (orally or topically), steroids (intranasal), antihistamines, expectorants, and other classes of drugs (e.g., herbal formulations). Instead of surgical intervention, pharmacological treatment and watchful waiting were implemented. Table 10 shows information about the different groups of drugs prescribed during the first visit and the recurrences (1st, 2nd, or 3rd recurrences). In the second column (1st visit) is information about the number of prescribed drugs for the 50 patients from the non-surgical group.

The number of different drugs prescribed after each recurrence may differ because each child had a different number of recurrences. The maximum number of prescribed drugs during one visit was four and the minimal was zero (visit without a prescribed drug). Most frequently, one drug was prescribed at one visit. On average, 1.72 drugs were prescribed at the first visit, 1.52 drugs at the first recurrence, 1.55 drugs at the second recurrence, and 2.14 drugs at the third recurrence (Table 11, Table 12, Table 13 and Table 14).

According to the medical histories of the children, most often, steroids were prescribed, irrespective of the type of visit or recurrence. Antibiotics were the second group of pharmacologic treatments prescribed during a recurrence (not including the 1st visit). The second group of pharmacotherapy included mucolytics. Among antibiotics, the most frequently prescribed were beta-lactam antibiotics, followed by macrolides. Topically (applied to the external auditory canal), a combination of aminoglycoside antibiotics with peptide antibiotics and steroids were administered.

## 4. Discussion

OME is one of the commonest conditions among children in their early years. In the US, USD 5 billion is spent annually in treating otitis media (OM) [30]. According to clinical data and the literature, screening for OME is not particularly useful [28,29]. Firstly, OME is usually asymptomatic and, from an epidemiological point of view, screening is not helpful due to the high incidence and recurrence in young children. Secondly, there is no marked difference in developmental outcomes (intelligence scores, behavioral and language problems) between children with and without screening. Finally, the self-limited nature of most episodes makes screening largely ineffectual. On the other hand, screening for OME is recommended at age 12–18 months for those children who have physical, sensory, or behavioral factors that make them liable for developing comorbidities. These factors are: (1) autism spectrum disorder and other pervasive developmental disorders; (2) craniofacial disorders that may lead to speech, language, or cognitive delays (e.g., Down syndrome); (3) blindness or other uncorrectable visual impairment; (4) permanent hearing loss independent of OME; (5) other confirmed or suspected speech or language disorders and delays; and (6) presence of cleft palate (with or without associated syndrome) [25]. Prevention of OM is based on a few strategies, a number of which are based on reducing modifiable risk factors, such as viral or bacterial infections and environmental risks [30]. According to the literature and clinical data, QoL in case of OME or OM are symptom scores, but they are not a QoL score. The difficulties in calculating a QoL are that OME is very common and may be mild. Therefore, in this analysis, QoL was not used as an independent measure. 

An additional point that deserves comment is the problem of antibiotic resistance, and this factor is challenging to assess. Over the last few decades, the problem of antibiotic resistance has been raised very often. Antibiotic resistance is the ability of microorganisms (e.g., bacteria) to develop resistance to an antibiotic to which they were once sensitive. There are two main species responsible for AOM, *Streptococcus pneumoniae* and *Haemophilus influenzae*, and both are becoming increasingly resistant to antibiotics; as a result, this may affect clinical treatment [31]. Moreover, antibiotic resistance affects society as a whole and calculating it in monetary terms is difficult. Children who are carriers of antibiotic-resistant bacteria will transmit these organisms to other members of their families. Consequently, a circle of transmission is created: the response to pharmacological agents decreases, antibiotic consumption increases, and this leads to growing resistance. The pace of invention of new antibiotics may not keep up, and, therefore, preventing AOM rather than treating it is considered of higher importance. The prevention of AOM is mostly based on immunization (viral and bacterial vaccines) and improvement in environmental factors [30,31]. In summary, the prescription of antibiotics should be avoided as far as possible [32].

Antibiotic resistance may be useful in discussing the benefits of the surgical approach and how tympanostomy with VTs and adenoidectomy can limit AOM incidents. For the entire study group (surgical group, *n* = 422), the number of healthy days ranged from 20 to 365, with a mean of 328.0 days (SD = 91.4) (Figure 1 and Figure 2). In the non-surgical group, the number of healthy days ranged from 13 to 365, with a mean of 169.2 days (SD = 127.3). The difference in the number of healthy days was statistically significant (*p* < 0.001). According to the statistics, in the 12-month observation period of the patients in the second group, there was no certainty of the effect of longer treatment outcome, due to small numbers. Of the 50 children, 10 had one year (365 days) without recurrence, but seven had less than 30 days without recurrence, and nine children were healthy for no more than 60 days. Due to greater numbers, the significance for treatment of the first group was higher than for the second, and the number of days without recurrence was higher than for the non-surgical group. According to the data from medical histories, antibiotics were the second commonest group of prescribed drugs (regardless of visit type—1st visit, 1st, 2nd, or 3rd recurrence), just behind nasal steroids.

According to the international consensus on the management of otitis media with effusion in children, which was published in 2018, non-surgical treatment in OME poorly addresses the underlying problem. There was a clear recommendation against using nasal steroids, antibiotics, antihistamines, or decongestants drug for the treatment of OME, due to not only cost issues but mainly because of side effects and a lack of convincing evidence of their long-term effectiveness. Additionally, the decision of inserting tympanostomy ventilation tubes (VTs) may be important as it can improve overall hearing difficulties and reduce the number of recurrent AOM episodes [25,31,32,33]. The same observations were obtained in our retrospective analysis. The number of recurrences of AOM in the non-surgical group was smaller. For the surgical group, the number of healthy days ranged from 20 to 365, with a mean of 328.0 days, and in the non-surgical group, the number of healthy days was lower and ranged from 13 to 365, with a mean of 169.2 days. The maximum number of prescribed drugs during one visit was four and the minimum was zero (visit without a prescribed drug). Most frequently, one drug was prescribed at one visit. On average, 1.72 drugs were prescribed at the first visit, 1.52 drugs at the first recurrence, 1.55 drugs at the second recurrence, and 2.14 drugs at the third recurrence. 

A limitation of this study is the number of the cases in both groups (surgical and non-surgical group), which was, respectively, 422 and 50, but, because this was a retrospective analysis, the authors had no control over the number of cases in each group. The starting point of data collection was different because the criteria of inclusion for each group were different in one point. In the surgical group, the starting point of the 12-month retrospective observation period was equal to the point after surgery. In the second group, the starting point of retrospective observation was the point of diagnosis, because, in this group, we were focused on the observation of these children without surgical intervention. The duration of observation in both groups was the same. 

## 5. Conclusions

The surgical approach (tympanostomy and the insertion of ventilation tubes with adenoidectomy) in children aged 1–6 years who have been diagnosed with otitis media with effusion is reasonable and beneficial for the child. This conclusion is based on two main reasons:

1. The surgery limits the recurrences of AOM during the following 12 months. Our data show that the average child from the control group (the non-surgical group) required approximately five-times more visits in the ensuing 12 months than the average child from the surgical group. The risk ratio (RR) of recurrence of AOM in the surgery group in comparison with the non-surgery group was equal to 0.21.
2. The surgery limits the prescription of steroids, antibiotics, mucolytics, antibiotics, and other drugs. On average, 1.72 drugs were prescribed at the first visit, 1.52 drugs were prescribed at the first recurrence, 1.55 drugs at the second recurrence, and 2.14 drugs at the third recurrence in this group. The reduction of prescribed antibiotics is important due to the rising resistance of bacteria to antibiotics.

## Figures and Tables

**Table 1:** Descriptive statistics for age in the surgical and non-surgical groups.

	*n*	Min	Max	M	SD
Surgical group	422	1.91	6.00	4.05	0.95
Non-surgical group	50	1.86	5.95	3.65	1.02

*n*, number of subjects; Min, minimal age; Max, maximum age; M, mean; SD, standard deviation.

**Table 2:** Sex distribution in the surgical and non-surgical groups.

	Male	Female	Total
Surgical group	250 (59.2)	172 (40.8)	422 (100.0)
Non-surgical group	26 (52.0)	24 (48.0)	50 (100.0)

Percentage is given in parentheses.

**Table 3:** Age distribution in the surgical and non-surgical groups.

	1–2 Years	2–3 Years	3–4 Years	4–5 Years	5–6 Years	Total
Surgical group	1 (0.2)	61 (14.5)	154 (36.5)	120 (28.4)	86 (20.4)	422 (100.0)
Non-surgical group	3 (6.0)	8 (16.0)	23 (46.0)	11 (22.0)	5 (10.0)	50 (100.0)

**Table 4:** The number of incidents of upper respiratory tract infection (URTI) in the medical history of children in the surgical and non-surgical groups.

	No	Yes	Total
Surgical group	233 (55.2)	189 (44.8)	422 (100.0)
Non-surgical group	28 (56.0)	22 (44.0)	50 (100.0)

“Yes” indicates confirmation of URTI in medical history; “No” indicates no confirmation of URTI in the medical history of the patient. Percentage is given in parentheses.

**Table 5:** The number of incidents of acute otitis media in the medical history of children in the surgical and non-surgical groups.

	No	Yes	Total
Surgical group	367 (87.0)	55 (13.0)	422 (100.0)
Non-surgical group	16 (32.0)	34 (68.0)	50 (100.0)

“Yes” indicates confirmation of AOM in medical history; “No” indicates no confirmation of AOM in the medical history of the patient. Percentage is given in parentheses.

**Table 6:** The distribution of data about the number of ENT specialist consultations in the surgical group related to otitis media prior to the surgery.

Number of ENT Consultations	Number of Patients	Percentage
1	312	73.9
2	63	14.9
3	29	6.9
4	8	1.9
5	5	1.2
6	2	0.5
7	1	0.2
8	1	0.2
9	1	0.2
Total	422	100.0

**Table 7:** The distribution of the number of AOM cases in 12- month follow-up period in the surgical and non-surgical groups.

	Number of ENT Consultations	Number of Patients	Percentage	Descriptive Statistics
Surgical group	0	312	73.9	Min = 0; Max = 3; M = 0.22; SD = 0.55
	1	63	14.9	
	2	29	6.9	
	3	8	1.9	
Non-surgical group	0	5	1.2	Min = 0; Max = 3; M = 1.16; SD = 0.91
	1	2	0.5	
	2	1	0.2	
	3	1	0.2	

**Table 8:** Descriptive statistics for the number of days without recurrence in the surgical and non-surgical groups.

	*n*	Min	Max	M	SD	Me
Surgical group	422	20	365	328.03	91.44	365
Non-surgical group	50	13	365	169.24	127.31	136.50

*n*, number of subjects; Min, minimal age; Max, maximal age; M, mean; SD, standard deviation; Me, median.

**Table 9:** Relative risk.

Relative Risk	0.2103
95% CI	0.1632 to 0.2710
Z statistic	12.060; *p* < 0.001
NNT (Benefit)	1.583
95% CI	1.347 (Benefit) to 1.918 (Benefit)

CI, confidence interval; NNT, number needed to treat.

**Table 10:** Information about the drugs prescribed at the 1st visit and at the 1st, 2nd, and 3rd recurrence.

How Many Different Pharmaceutical Groups of Drugs Were Prescribed?	1st Visit (Time of Diagnosis of OME)	1st Recurrence during 12-Month Observation Period	2nd Recurrence during 12-Month Observation Period	3rd Recurrence during 12-Month Observation Period
0	4	3	2	-
1	20	18	4	1
2	15	15	2	4
3	8	3	3	2
4	3	1	-	-

**Table 11:** Information about the drugs prescribed at the 1st visit.

Antibiotics		Steroids		Antihistamines		Mucolytics		Other	
Name of Substance	Quantity	Name of Substance	Quantity	Name of Substance	Quantity	Name of Substance	Quantity	Name of Substance	Quantity
Amoxiciline + clavulonic acid	6	Fluticasone furoate	8	Desloratadine	2	Ambroksol hydrochloride	10	Oxymetazolini	2
Neomycin + gramicidin + fludrocortisone	1	Fluticasone propionate	8	Levocetirizine	4	Bromhexine hydrochloride	2	Xylometazoline Hydrochloride	3
Cefuroxime axetil	2	Mometasone furoate	21	Cetirizine	3	Carbocisteine	2	Herbal medicines	2
Tobramycin	2	Budesonide	3					Trimethoprim/sulfamethoxazole	1
Total:	11		40		9		14		8

**Table 12:** Information about drugs prescribed at the 1st recurrence.

Antibiotics		Steroids		Antihistamines		Mucolytics		Other	
Name of Substance	Quantity	Name of Substance	Quantity	Name of Substance	Quantity	Name of Substance	Quantity	Name of Substance	Quantity
Amoxicilin + clavulonic acid	8	Fluticasone furoate	5	Desloratadine	4	Ambroxol hydrochloride	5	Oxymetazolini hydrochloridum	7
Amoxicillin	1	Fluticasone propionate	3	Levocetirizine	2	Bromhexine hydrochloriudum	1	Xylometazoline Hydrochloride	1
Cefuroxime axetil	2	Mometasone furoate	13			Carbocisteine	1	Hydrocortisone + Oxytetracycline hydrochloridum + Polymyxin B	1
Tobramycin	1	Budesonide	2			Acetylcysteine	1		
Neomycin + gramicidin + fludrocortisone	1								
Azithromycin	1								
Total:	14		23		6		8		9

**Table 13:** Information about drugs prescribed at the 2nd recurrence.

Antibiotics		Steroids		Antihistamines		Mucolytics		Other	
Name of Substance	Quantity	Name of Substance	Quantity	Name of Substance	Quantity	Name of Substance	Quantity	Name of Substance	Quantity
Amoxicilin + clavulonic acid	2	Fluticasone furoate	1	Desloratadine	1			Xylometazoline Hydrochloride	1
Cefuroxime axetil	2	Fluticasone propionate	1	Levocetirizine	1			Hydrocortisone + Oxytetracycline hydrochloridum + Polymyxin B	1
		Mometasone furoate	6						
		Budesonide	1						
Total:	4		9		2		0		2

**Table 14:** Information about drugs prescribed at the 3rd recurrence.

Antibiotics		Steroids		Antihistamines		Mucolytics		Other	
Name of Substance	Quantity	Name of Substance	Quantity	Name of Substance	Quantity	Name of Substance	Quantity	Name of Substance	Quantity
Amoxicilin + clavulonic acid	1	Fluticasone furoate	1					Oxymetazolini hydrochloridum	2
Cefuroxim axetil	2	Mometasone furoate	5						
Azithromycin	1	Budesonide	1						
Neomycin + gramicidin + fludrocortisone	1								
Total:	5		7		0		0		2

## Data Availability

The data presented in this study are available on request from the corresponding author. The data are not publicly available due to protection of personal medical data.
